# Early Holocene greening of the Sahara requires Mediterranean winter rainfall

**DOI:** 10.1073/pnas.2024898118

**Published:** 2021-05-31

**Authors:** Rachid Cheddadi, Matthieu Carré, Majda Nourelbait, Louis François, Ali Rhoujjati, Roger Manay, Diana Ochoa, Enno Schefuß

**Affiliations:** ^a^Institut des Sciences de l’Évolution de Montpellier, CNRS, Institut de Recherche pour le Développement, University of Montpellier, 34000 Montpellier, France;; ^b^Institut Pierre-Simon Laplace-Laboratoire d'Océanographie et du Climat: Expérimentations et approches numériques, CNRS, Institut de Recherche pour le Développement, Muséum National d’Histoire naturelle, Sorbonne Université (Pierre and Marie Curie University), 75006 Paris, France;; ^c^Centro de Investigaciòn Para el Desarrollo Integral y Sostenible, Laboratorios de Investigación y Desarrollo, Facultad de Ciencias y Filosofia, Universidad Peruana Cayetano Heredia, Lima 15102, Peru;; ^d^LGMSS, URAC45, University Chouaib Doukkali, El Jadida 24000, Morocco;; ^e^UR-SPHERES, University of Liège, 4000 Liège, Belgium;; ^f^Laboratoire Géoressources, URAC42, Université Cadi Ayyad, Marrakech 40000, Morocco;; ^g^MARUM - Center for Marine Environmental Sciences, University of Bremen, 28359 Bremen, Germany

**Keywords:** African humid period, green Sahara, Holocene, paleoclimate reconstructions, vegetation model simulations

## Abstract

The greening of the Sahara, associated with the African Humid Period (AHP) between ca. 14,500 and 5,000 y ago, is arguably the largest climate-induced environmental change in the Holocene; it is usually explained by the strengthening and northward expansion of the African monsoon in response to orbital forcing. However, the strengthened monsoon in Early to Middle Holocene climate model simulations cannot sustain vegetation in the Sahara or account for the increased humidity in the Mediterranean region. Here, we present an 18,500-y pollen and leaf-wax δD record from Lake Tislit (32° N) in Morocco, which provides quantitative reconstruction of winter and summer precipitation in northern Africa. The record from Lake Tislit shows that the northern Sahara and the Mediterranean region were wetter in the AHP because of increased winter precipitation and were not influenced by the monsoon. The increased seasonal contrast of insolation led to an intensification and southward shift of the Mediterranean winter precipitation system in addition to the intensified summer monsoon. Therefore, a winter rainfall zone must have met and possibly overlapped the monsoonal zone in the Sahara. Using a mechanistic vegetation model in Early Holocene conditions, we show that this seasonal distribution of rainfall is more efficient than the increased monsoon alone in generating a green Sahara vegetation cover, in agreement with observed vegetation. This conceptual framework should be taken into consideration in Earth system paleoclimate simulations used to explore the mechanisms of African climatic and environmental sensitivity.

## Significance

Explaining the greening of the Sahara during the Holocene has been a challenge for decades. A strengthening of the African monsoon caused by increased summer insolation is usually cited to explain why the Sahara was vegetated from 14,000 to 5,000 y ago. Here, we provide a unique climate record of quantified winter, spring, and summer precipitation in Morocco over the past 18,500 y, and numeric simulations, which show that moisture contributions from the Mediterranean Sea and the North Atlantic Ocean in winter, were as important as the expanded summer monsoon for the greening of the Sahara during the African humid period. The findings of this study will help to better understand and simulate climate variability over northern Africa.

Moisture availability in northern Africa, from the Sahel to the Mediterranean coast, is a critical issue for both ecosystems and human societies yet represents one of the largest uncertainties in future climate simulations (1, 2). The humid time span in the African Sahel and Sahara, known as the African humid period (AHP) (3–13), occurred in northern Africa after the last glacial period (4, 10, 11, 14–16) and lasted from ca. 14.5 to 5 ky ago (ka), with an optimum between 11 and 6 ka (11, 16). This prominent climatic event allowed semiarid, subtropical, and tropical plant species to spread outside their modern ranges (14) into the Sahara and human populations to inhabit what is known as the green Sahara (5, 17).

The green Sahara is an example of extreme environmental change, which highlights the region’s extraordinary sensitivity and the need to better understand its hydroclimatic variability. Current explanations for the greening of the Sahara point to the Earth’s orbital changes during the Early Holocene, leading to increased boreal summer (JJA) insolation, which drove the intensification and northward expansion of the JJA monsoon over northern Africa (15, 18), aided by strong positive feedbacks from the land surface (19–22). Reproducing the green Sahara has posed a lasting challenge for climate modelers. The influence of the African monsoon extends only to ∼24° N (with or without interactive vegetation) in most Middle Holocene simulations, which is insufficient to sustain a vegetated Sahara. Models that integrate vegetation, dust, and soil feedbacks push the monsoon influence further north but still have discrepancies with proxy data (18, 23, 24).

When all surface feedbacks are prescribed, simulated precipitation in the northern Sahara is still too low compared to paleoclimatic evidence for substantially increased moisture at 31° N (11, 13) or too high in the 15 to 20° N range (20), creating incompatibility with prescribed vegetation (22). Additional sources of moisture (25, 26) may have contributed to an AHP that extended toward the Mediterranean borderlands through different mechanisms. However, identifying the moisture sources over North Africa during the AHP requires paleoclimate records of both winter (DJF) and JJA precipitation.

**Fig. 1.**
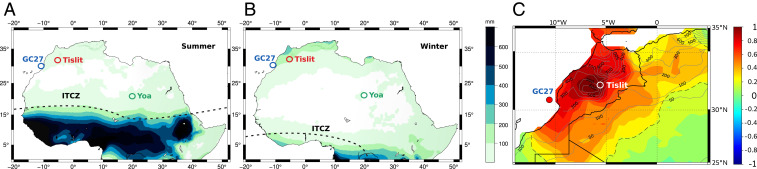
Maps showing the location of Lake Tislit, core GC27 (11), and Lake Yoa (6), along with the schematic position of the Inter Tropical Convergence Zone, with modern mean JJA (*A*) and DJF (*B*) rainfall (56). Map *C* shows the correlation coefficients (*r*) between Tislit and northern Morocco for DJF precipitation variability over the 1901 to 2010 time period (using the 20th century reanalysis of National Oceanic and Atmospheric Administration; https://psl.noaa.gov/data/20thC_Rean/). The limit of statistical significance (0.05 level) is shown by the dashed black line. Gray contours indicate annual precipitation isohyets (millimeter/year).

In the High Atlas Mountains, we collected an 8.5-m sediment core from Lake Tislit (ca. 32° N). The lake traps pollen grains from the surrounding landscape and, as a closed lake, is highly sensitive to hydroclimate fluctuations. It is ideally located for capturing the climatic variability of the Mediterranean and northwestern Sahara (Fig. 1). The Tislit sequence yielded unique hydrological data from leaf-wax stable isotopes and ostracod stable oxygen isotopes (δ^18^O), as well as a quantified time series of seasonal rainfall from the fossil pollen assemblages. Based on the findings from the Tislit record, we propose a precipitation regime for the AHP, including both Mediterranean DJF precipitation and monsoon JJA precipitation increases. Using a dynamic vegetation model for a conceptual experiment with 9 ka boundary conditions, we evaluate how a change in the seasonal distribution of precipitation over the Sahara can affect its revegetation.

## Results and Discussion

**Fig. 2.**
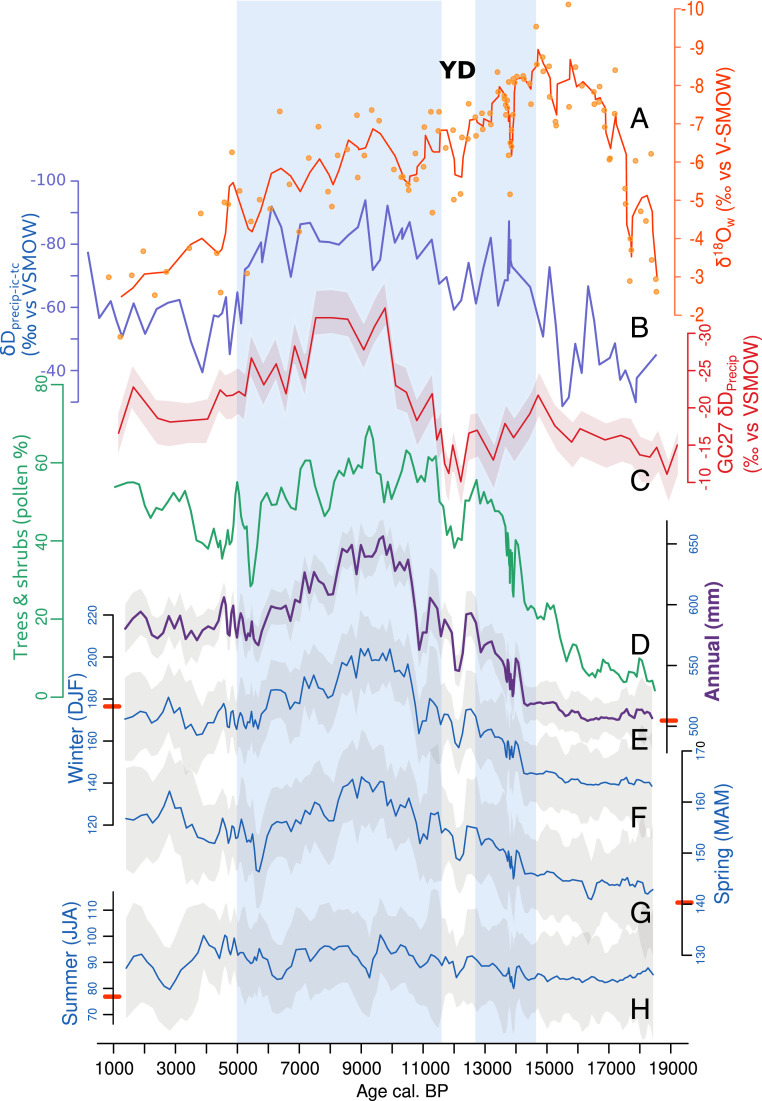
Time series of vegetation and climate proxies obtained from Lake Tislit and δD_precip_ from core GC27, located offshore of Morocco (11). (*A*) Lake water δ^18^O calculated from fossil ostracods δ^18^O. (*B*) δD_precip_ (corrected for ice volume and temperature effects; see *Materials and Methods* and *SI Appendix*, Fig. S6). (*C*) δD_precip_ of core GC27 off the coast of Morocco (11). (*D*) Pollen percentages of trees and shrubs. Pollen-based reconstructions of annual (*E*), DJF (*F*), spring (MAM) (*G*), and JJA (*H*) precipitation in millimeters, with uncertainty values that correspond to the maximum and minimum values obtained using a jackknife procedure (gray area). All Tislit curves correspond to moving averages over three samples. The points in *A* correspond to raw data. The red bars on the *y*-axes of the pollen-based reconstructions (*E*–*H*) indicate the modern values. The AHP is highlighted in light blue shading. YD, Younger Dryas; VSMOW, Vienna Standard Mean Ocean Water.

From the Tislit sediment core, we obtained a continuous, radiocarbon-dated record of the last 18,500 y (*SI Appendix*, Fig. S1). Its pollen content (*SI Appendix*, Fig. S2) shows a general predominance of Mediterranean ecosystems, with arid steppe elements (*Artemisia* and *Chenopodiaceae*), between 18.5 and 15 ka, and sclerophyllous evergreen tree and shrub taxa (*Pinus*, *Quercus*, *Olea*, and *Pistacia*) during the AHP. After 5 ka, the landscape continued to be dominated by pine and oak trees, with recolonization by the steppic sagebrush. Mediterranean plant species such as *Olea* and *Quercus* evergreen are physiologically adapted to JJA drought (27–29) and grow in areas with marked seasonal distribution of precipitation over the year (*SI Appendix*, Fig. S3). The spread of these and other Mediterranean plant taxa to Tislit during the Holocene (*SI Appendix*, Fig. S2) indicates higher precipitation in DJF than in JJA. The Tislit pollen record allowed us to quantitatively reconstruct the seasonal changes in temperature (*SI Appendix*, Fig. S4) and precipitation (Fig. 2) in northern Africa using the weighted median of the climatic ranges of the pollen taxa (see *Materials and* *Methods*). These climatic ranges were obtained from an extensive database of georeferenced modern plant distributions. The accuracy of the method was validated for Morocco by comparing precipitation reconstructed from a modern pollen dataset to instrumental values (*SI Appendix*, Fig. S5; see *Materials and* *Methods*).

The pollen-based reconstruction shows that JJA rainfall (Fig. 2*H*) has not changed significantly over the past 18,500 y, while DJF (Fig. 2*F*) and spring (Fig. 2*G*) rainfall increased by ∼30% between ca. 14 and 9.5 ka, reaching their highest values between ca. 10.5 and 8.5 ka, which corresponds to the AHP, as evidenced in northern African sites (10). DJF rainfall decreased then by ∼15% by ca. 5.7 ka. This result confirms and quantifies the inference based on the ecological and physiological requirements of the fossil plant taxa. The increase in annual precipitation during the AHP (Fig. 2*E*) is thus robustly related to a higher contribution from DJF and spring rainfall rather than to JJA rainfall, which remained almost unchanged through the whole period (Fig. 2*H*). The effect of temperature on ecosystems through evaporation and water availability is considered to be minor since evaporation is low in the DJF rainfall season, and Mediterranean vegetation is adapted to JJA drought.

δ^18^O of ostracod shells from the Tislit core were also analyzed and corrected for temperature and vital effects to obtain water lake δ^18^O values (see *Materials and* *Methods*). Because Lake Tislit is a closed basin, the water lake δ^18^O record reflects variations in the lake’s hydrological budget (Fig. 2*A*). Highly depleted δ^18^O values observed between 18 and 13 ka indicate a massive increase in freshwater input, resulting from the melting of a nearby glacier that stood close to the lake (30). After the disappearance of the glacier at ca. 12 ka (30), δ^18^O variations reflect changes in precipitation–evaporation and suggest humid conditions in the Early Holocene, followed by a progressive aridification that is consistent with the pollen record.

Stable hydrogen isotope compositions (δD) in leaf wax were measured (see *Materials and* *Methods* and *SI Appendix*, Fig. S6) to assess precipitation isotope (δD_precip_) changes (Fig. 2*B*) during the plant growing season (31). Given that it is more directly linked to precipitation and not as influenced by evaporation, there are substantial differences between the leaf-wax δD record and the ostracod δ^18^O record. However, the former does clearly record a period with isotopically depleted precipitation that is consistent with increased rainfall between 11 and 5 ka.

Although the annual pollen-derived precipitation estimates and the δD_precip_ reconstructions in the Tislit record are in broad agreement, the enrichment in δD_precip_ at 5 ka (Fig. 2*B*) appears more abrupt than the pollen-based precipitation reconstruction. This may suggest that δD_precip_ in Tislit was not driven solely by changes in precipitation amount. Morocco currently has three main moisture sources: the distal North Atlantic Ocean, the proximal North Atlantic Ocean, and the Mediterranean Sea (32). The distal North Atlantic moisture source provides the isotopically most depleted rainfall, followed by the proximal North Atlantic and the Mediterranean source, with the latter being isotopically most enriched (32). Thus, a plausible explanation for the relatively abrupt δD_precip_ change at 5 ka (Fig. 2*B*) would be the sudden cooling in the North Atlantic Ocean at that time (33), which would have reduced the relative influence of the distal North Atlantic, isotopically most depleted moisture source in favor of other more isotopically enriched moisture sources.

### Sources of AHP Moisture in Northern Sahara.

An analysis of precipitation variability over the period 1901 to 2010 in Morocco shows significant coherence of DJF precipitation between Tislit and a broader adjacent area that includes coastal areas and extends into western Algeria and northern Sahara to 25° N (Fig. 1*C*). The paleoclimate record of Tislit is thus likely not a local feature and representative of the climate history of a wider region. In addition, there is a strong coherence between the δD_precip_ record from Lake Tislit (Fig. 2*B*) and the δD_precip_ record of marine core GC27 (Fig. 2*C*) from offshore Morocco (11) (Fig. 1), with regards to the timing of the two d-depleted peaks at 15 to 12.7 ka and 11 to 5 ka, which are separated by a relatively drier episode during the Younger Dryas. This double-peak signal is also observed further south in the Sahel and in Equatorial Africa (10, 11, 34). Considering the proximity of core GC27—located at about the same latitude but ∼450 km away from Lake Tislit—the similarity of their leaf-wax δD records, and the fact that Tislit precipitation variability is strongly correlated with that in the catchment that contributed sediments to core GC27 (Fig. 1*C*), both sites most likely recorded a similar climate regime. Consequently, the increased precipitation recorded during the AHP in the GC27 marine core, and possibly in the CG37 core located at 27° N (11), was not associated with the African JJA monsoon but rather with the DJF and spring rainfall, as it is for Lake Tislit. In both the Mediterranean and the monsoonal climate system, the similarity of the δD_precip_ records was probably caused by an increase in precipitation amount in parallel with the prevalence of a remote moisture source (i.e., the northern Atlantic Ocean versus the Gulf of Guinea, respectively).

Numeric simulations of the Earth’s climate forced by precession changes have shown that during periods of maximum boreal JJA insolation and minimum boreal DJF insolation, such as the Early Holocene, the equator-to-pole temperature gradient is weaker in DJF, which is associated with a southern shift of the Hadley cell, the westerlies, and the Mediterranean storm track, bringing increased DJF precipitation to northern Africa (26). Although simulations show this mechanism to have a relatively small rainfall effect in northern Africa (11, 35), it is qualitatively consistent with our data. The in-phase variability of Mediterranean DJF rainfall with the North African JJA monsoon during precessional cycles has also been identified in paleoclimate records from the northern Mediterranean (36). We suggest that the strengthened Mediterranean DJF storm track reached northern Africa as a response to lower DJF insolation and may have been the moisture source for the increased DJF rainfall in the northern Sahara during the Holocene AHP.

### An Alternative Precipitation Regime to Explain the Green Sahara.

As the Tislit record shows, the monsoon did not reach the northernmost Sahara. The Mediterranean DJF rainfall zone and the African monsoon zone extended southward and northward, respectively, and may have overlapped in the Sahara, maintaining a vegetation cover. Below, we propose a hypothetical scenario of this theoretical framework to test its implications for the greening of the Sahara.

Today, the African monsoon reaches ∼15° N (37), while the Mediterranean DJF rainfall zone is limited to a narrow band close to the Mediterranean coast (Fig. 1). These two rainfall zones are separated by a vast desert that receives less than 100-mm rain per year (38). Fossil pollen data indicate that the green Sahara was composed of a steppe biome north of 24° N and a mix of steppe and Sahelian biomes from 15 to 24° N (3, 39). In Middle and Early Holocene simulations, the maximum latitude reached by the African monsoon is generally 24° N, if we exclude sensitivity experiments that prescribed extended vegetation (21). In climate simulations, orbitally paced humid periods such as the Early Holocene are associated with intensified westerlies in northern Africa and the occurrence of low-pressure anomalies as far south as 18° N (36, 40). We therefore propose, as a hypothesis, that the influence of the DJF storm track shifted south during the AHP to reach at least 24° N and potentially 18° N. This means that an environment characterized by two rainfall seasons would have existed during the AHP between 18 and 24° N. The total annual precipitation in this zone would be small, given its location at the boundary of both precipitation systems. However, even with low rainfall, two rainy seasons would have a major impact on ecosystems by shortening the dry season to less than 6 mo instead of 10 to 11 mo. The length of the dry season in arid regions has a direct impact on ecosystems (41, 42). This effect of a double rainy season on vegetation would be enhanced by positive feedbacks from the vegetation and soil (15, 20, 22).

### Testing Green Sahara Precipitation Regimes with a Vegetation Model.

**Fig. 3.**
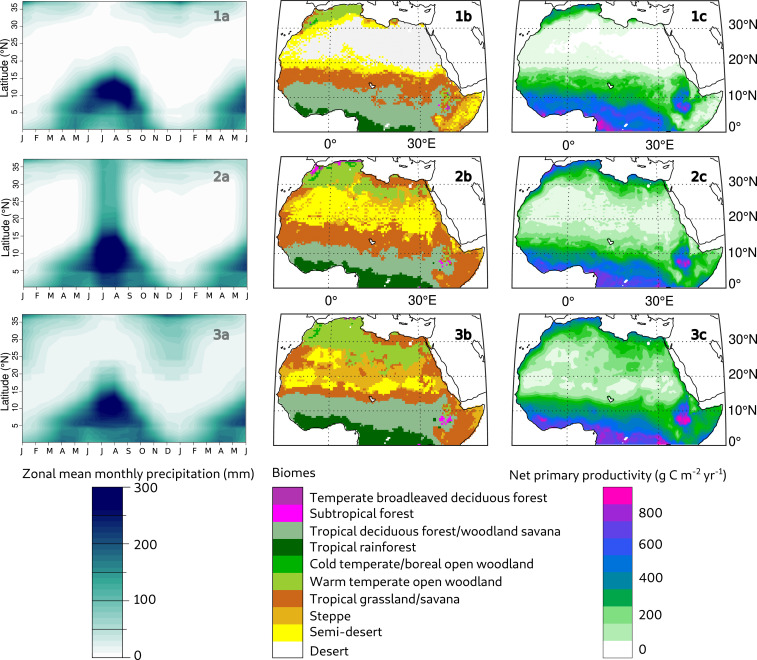
Zonal mean monthly precipitation (*1a*, *2a*, *3a*), used for simulating biomes (*1b*, *2b*, *3b*) and NPP (*1c*, *2c*, *3c*) at 9 ka with the CARAIB vegetation model. The three simulations were performed using the following: 1) HadCM3 9 ka climatology (47); 2) 300-mm precipitation added each year to modern values, only during the monsoon season, over the whole simulated area; and 3) an increase of 300-mm precipitation each year below 18° N in the summer season only, above 24° N in the DJF season only, and with a progressive transition between 18 and 24° N, where precipitation occurs in both JJA and DJF (*SI Appendix*, Table S2). The data for simulations 2 and 3 are available in the *SI Appendix*.

We tested whether the hypothesis outlined above can maintain a green Sahara in the dynamic vegetation model CARAIB (Carbon Assimilation in the Biosphere) (see *Materials and* *Methods*), whose performance is well demonstrated (43–46). Biomes and net primary productivity (NPP), produced in a control simulation forced with modern climate data, correctly reproduce the vegetation observed today in northern Africa (*SI Appendix*, Fig. S7). Three different precipitation regimes were tested as inputs into the vegetation model under 9 ka insolation and atmospheric CO_2_ conditions (Fig. 3). In simulation 1, CARAIB was forced by the rainfall regime, produced by the Hadley Centre Coupled Model version 3 (HadCM3) model paleoclimate simulation at 9 ka, and corrected for its modern biases (47) (Fig. 3, *1a*). Simulation 1 is an illustrative example of climate model simulations of the Early Holocene. Simulations 2 and 3 use idealized scenarios as inputs to test the effect of seasonal rainfall distribution on Sahara biomes and NPP at 9 ka, where 300 mm/y was added to the modern instrumental precipitation regime over the whole of northern Africa, with two different seasonal distributions. The rainfall increase of 300 mm/y lies within the range of quantitative estimates derived from fossil pollen records in the green Sahara (48, 49) but is likely an underestimation in the Sahel (14 to 18° N). The additional annual precipitation was distributed in the JJA season in simulation 2 as a simplified representation of the hypothesis that the AHP was related solely to a strengthened African monsoon (Fig. 3, *2a*). Simulation 3 is a test of the alternative model proposed, in which the additional 300 mm/y was seasonally distributed from north to south to represent a gradual DJF rainfall penetration down to 18° N, combined with a gradual northward expansion of the JJA monsoon up to 24° N, thus including an overlap zone with a weak double rainy season between 18 and 24° N (Fig. 3*a* and *SI Appendix*, Table S2).

The first simulation yielded vast semidesert or desert biomes (Fig. 3, *1b*) across the whole Saharan belt, with an NPP under 50 g ⋅ C ⋅ m^−2^ ⋅ yr^−1^ (Fig. 3, *1c*). The precipitation regime produced by the HadCM3 model at 9 ka fails to sustain a green Sahara, in line with most climate system models (22). Simulations 2 and 3 both show higher NPP as expected but with striking differences that confirm the strong effect of rainfall seasonality on vegetation in the Saharan belt.

In the monsoon-only scenario (simulation 2), the Sahara is still largely dominated by a semidesert biome (Fig. 3, *2b*), with NPP values below 50 g ⋅ C ⋅ m^−2^ ⋅ yr^−1^ (Fig. 3, *2c*), despite the additional 300 mm of rainfall. This is neither compatible with the vegetation reconstructed at 6 ka in Lake Yoa (6) nor with evidence of pastoralism across the Sahara in the Early Holocene (5). In addition, subtropical biomes appear close to the Mediterranean coast in Morocco, which is also in contradiction with our pollen record and others from a lower elevation in the Middle Atlas Mountains (50, 51). In contrast, with the same annual rainfall, the sensitivity test combining Mediterranean DJF rainfall and JJA monsoon rainfall (Fig. 3, *3a*) yields higher NPP values (Fig. 3, *3c*) and biomes (Fig. 3, *3b*) that are consistent with observations (3, 14, 39). In this simulation, the semidesert is reduced to patches and several vegetation corridors that were necessary for plant species migration (14) are present.

The more plausible green Sahara produced by vegetation simulation 3 supports the strengthening and southward shift of the Mediterranean DJF rainfall zone inferred from the Tislit record. It also provides evidence that enhanced DJF rainfall over the northern Sahara, coupled with the enhanced African monsoon, was necessary for the green Sahara to persist over a period of several millennia.

## Conclusions

In this study, we present a paleoclimate record with quantified DJF, spring, and JJA precipitation from Lake Tislit in Morocco (32° N), which provides a constraint on the northward expansion of the African monsoon commonly invoked to explain the AHP in northern Africa between 14.5 and 5 ka. Using a vegetation model hypothesizing an N-S gradient of DJF and JJA precipitation overlapping between 18 and 24° N, we showed that Saharan vegetation is denser and closer to observation than in a monsoon-only scenario. Although the additional rainfall used to simulate green Sahara was small, its annual bimodal occurrence reduced the length of the dry season and increased soil water availability, with de facto effects on plant primary productivity, biomass, and positive albedo feedbacks to climate.

Additional paleoclimate records of DJF and JJA precipitation during the AHP are needed, especially between 18 and 24° N, to track the latitudinal position of the different precipitation systems. This conceptual framework represents an important shift in the target for evaluating Earth system models and their ability to simulate African climate variability (52, 53).

## Materials and Methods

### The Geographical Setting of the Tislit Record.

Morocco is located in Northwest Africa and has vast coastal areas, a mountainous interior (the Atlas Mountains), and large deserted regions that extend south. The climate is Mediterranean, with DJF moisture transported via storm tracks from the Atlantic Ocean and the Mediterranean Sea, and a dry JJA season dominated by Saharan air masses. Lake Tislit extends about 1.2 km E-W and 600 m N-S in a tectonic pull-apart basin that was formed within Jurassic red beds (54) located south of the highest elevations of the central High Atlas. Today, the lake is mainly fed by snowmelt during spring. Evaporation is high during JJA, which leads to a lake-level change of several meters over the course of the year, but the annual precipitation to evaporation budget is still positive, and the lake is permanent. The hills surrounding the lake are treeless except for few stands of pines (*Pinus halepensis*) and poplars (*Populus alba*) on the edge of the lake. The dominant vegetation is grass. The local human impact on vegetation is minor because of the very low number of inhabitants at the elevation of the lake.

In 2016, we collected an 8.5-m–long sediment core (32°11′ N 5° 38′ W, 2,250-m above sea level), under a 5-m water column in the northeast part of Lake Tislit, using a Russian corer.

### Pollen-Based Climate Reconstruction.

Fossil pollen data from temperate ecosystems with a markedly seasonal climate, such as the Mediterranean, are a powerful climate proxy for evaluating past seasonal changes in precipitation. As an illustration from the Tislit pollen data, herbs or trees such as *Asphodelus* or *Olea* require low levels of precipitation during JJA (less than 50 mm in JJA) but require more than 300 mm in DJF (*SI Appendix*, Figs. S3 and S8). Their occurrence in the pollen record constrains past seasonal precipitation levels. In the rest of this section, we describe the quantitative reconstruction process and its uncertainties.

The first step was the assignment of fossil pollen taxa to modern plant species. Pollen grains are often identified at the family or genera level because most species of the same genus produce pollen grains that are morphologically indistinguishable. Drawing on our background in palynology, botany, and ecology, we assigned 45 of the pollen taxa identified in the Tislit record (*SI Appendix*, Table S1) to plant species that are available in a database, containing 864 georeferenced distributions obtained from *Flora Europaea* (55–57) and the Global Biodiversity Information Facility (58). The remaining fossil pollen taxa (33) were not included in the climate reconstruction because they were either aquatic, human related, or less than 1% of the total pollen sum. Pollen grains are mainly dispersed by wind (pollen grains from entomophilous plant species are rare in the fossil samples), meaning that some may be transported over long distances and affect the reconstruction. These pollen grains usually do not exceed 1% of the total pollen sum, which is the threshold we used to select pollen taxa and avoid this error source in the climate reconstruction.

The geographical occurrence of plants was crossed with modern interpolated climatology from the WorldClim database (59) to estimate the temperature and precipitation ranges of the fossil taxa in each season. Changes in the contribution of species within a pollen taxon do not affect the reconstruction, since the pollen taxon’s climatic range always includes the species climatic range.

The fossil pollen samples contain different numbers and types of taxa. For each fossil sample, monthly precipitation values were reconstructed by the weighted median of the pollen taxa precipitation medians using pollen percentages as weights. The reconstruction uncertainty is estimated using a jackknife procedure, leaving a taxon out at each iterative climate estimation, as many times as the number of taxa in each fossil sample. This quantification method is based on the assumption that the modern climatic niche of taxa is representative of their niche in the past millennia and that taxa are more abundant when climate is close to the optimum of their niche.

The small variation in atmospheric CO_2_ during the Holocene (roughly between 250 to 270 ppmv) (60) had less impact on the ecosystems than during the Last Glacial Maximum (61) and should therefore represent a negligible bias in the reconstructed precipitation values during the AHP.

We validated the pollen-based climate reconstruction method with a modern dataset of pollen samples collected in Morocco (62) (*SI Appendix*, Fig. S5). The climate values reconstructed from the modern pollen samples were compared to the instrumental climatology of WorldClim (59). All reconstructed precipitation values correlated well (*r* > 0.6) with the observed climate. Precipitation is generally overestimated in arid areas where all plants species are in the limit of their range, while the method considers species’ optimum climate value to be the most likely estimate. Thus, the pollen-based climate reconstruction approach should be used with caution in areas where annual precipitation is lower than ca. 300 mm/y. Tislit, however, lies in the climatic range that is not affected by this bias (*SI Appendix*, Fig. S5).

All statistics and database queries were performed using R software version 3.6.3 (63) with Akima (64), RMySQL (65), and Stats libraries (63).

### Oxygen Isotopes from Ostracod Shells.

Of the 171 samples collected from the Tislit core, 142 samples of 1-cm^3^ sediment were disaggregated in deionized water with detergent, then rinsed, sieved, and dried. Ostracod shells were picked out, cleaned a second time by ultrasonication in methanol (MeOH), and dried. Dry ostracod remains (mostly disarticulated carapace valves and their larger fragments) were identified under a microscope at up to 500× magnification using taxonomy identification keys (66).

When possible, 30 ostracod shells of the last four (fifth to eighth) juvenile stages (mostly 0.49 to 0.83 mm in length) of a species belonging to the genus *Candona* were picked out for isotopic analysis. This genus was selected for its abundance throughout the sediment core. By using the same taxon for isotopic analyses in the whole record, we avoided variations due to species-specific isotopic offsets (67). Ostracod shells are composed of calcite. Scanning electronic microscope observations showed excellent preservation. Only 13 samples out of 142 did not have enough valves for isotopic analysis. The samples were dissolved in saturated phosphoric acid at 70 °C in a Kiel IV carbonate device. The oxygen isotopic composition of the CO_2_ obtained was measured in a Thermo Delta V mass spectrometer. The analytic reproducibility (±1σ) was 0.1‰ based on long-term analysis of standard laboratory calcite samples. Isotopic values (Fig. 2*A*) are reported in permil as deviations relative to the Vienna Pee Dee Belemnite international standard. Ostracod isotopic values were corrected from the biological fractionation offset of +2.2‰ estimated for *Candoninae* ostracods (67). The lake water δ^18^O was estimated using the paleotemperature equation of Kim and O’Neil (68) for inorganic calcite and the pollen-derived annual temperature values (*SI Appendix*, Fig. S4).

### Leaf-Wax Analyses.

For the leaf-wax analyses, we used 89 samples from the Tislit core. A total of 2 to 3 g of dried and finely ground sediment samples were extracted in an ASE200 accelerated solvent extractor using a dichloromethane (DCM):MeOH 9:1 solution, at 1,000 psi and 100 °C, for three cycles lasting 5 min each. Known amounts of squalane were added prior to extraction as an internal standard. The total lipid extracts (TLEs) were dried in a Heidolph rotary evaporator system. Residual water was removed over columns of Na_2_SO_4_ using hexane as an eluent. The TLEs were saponified in 0.5 mL of 0.1 M KOH in MeOH solution. After adding bidistilled water, the neutral fractions were obtained by liquid–liquid extraction using hexane. The neutral fractions were separated over pipette columns of deactivated silica (1% H_2_O) using hexane, DCM, and DCM:MeOH 1:1 as eluents to obtain apolar, ketone, and polar fractions, respectively. The apolar fractions were further purified by cleaning them over columns of AgNO_3_-coated SiO_2_ using hexane as a solvent to remove unsaturated compounds. *n-*alkanes were quantified using a Thermo Fisher Scientific Focus gas chromatograph equipped with a 30-m Rxi-5ms column (30 m, 0.25 mm, and 0.25 μm) and a flame ionization detector. Quantification was achieved by comparing the integrated peak areas to external standard solutions, consisting of *n*-alkanes of varying chain lengths. Repeated analyses of standard solutions indicated a quantification precision of 10%. All samples were dominated by odd-numbered, long-chain *n*-alkanes, with *n*-C_29_ and *n*-C_31_ alkanes being the most abundant homologs in all samples. The carbon preference index values (69) of long-chain *n*-alkanes were 7.8 on average (3.6 to 15.4), indicating their origin in epicuticular waxes of terrestrial higher plants (70). δ^13^C and δD analyses of *n-*alkanes are detailed in *SI Appendix*.

### Vegetation Model Simulations.

Vegetation was simulated with the CARAIB (43–45) Dynamic Vegetation Model (*SI Appendix*). A control simulation was run with 280 ppmv of atmospheric CO_2_ (*SI Appendix*, Fig. S7) using climate data from the Global Soil Wetness Project Phase 3 (GSWP3) of the 1901 to 1930 period instead of the preindustrial, which lacks gridded climatological data. This dataset covers the whole 20th century. It is based on the 20th Century reanalyses (prepared by the National Oceanic and Atmospheric Administration, see https://www.psl.noaa.gov/data/20thC_Rean/), and the meteorological data are provided with a daily time step. The GSWP3 data used in the 1901 to 1930 control climate provided a 30-y sequence of daily values for each of the model’s input climate variables. After a spin-up phase, the CARAIB model was run over this 30-y series and an average was calculated, before analyzing the results and plotting biomes and NPP maps.

In simulation 1 (Fig. 3, *1*), the climatic anomalies between 9 and 0 ka from HadCM3 were computed, interpolated, and added to the 1901 to 1930 climate series to obtain a 30-y climate series for 9 ka at 0.5° × 0.5°. CARAIB was run using this climate series as an input together with an atmospheric CO_2_ of 263 ppmv characteristic of the Early Holocene (60). To calculate the solar flux in CARAIB, 9 ka orbital parameters were used together with the percentage of sunshine hours.

For simulations 2 and 3 (Fig. 3, *2* and *3*), two 30-y precipitation series were artificially constructed, by adding 300 mm/y of precipitation to the 1901 to 1930 series, uniformly over the whole simulated area. In simulation 2, the 300 mm were distributed over the JJA months only (June to September; *SI Appendix*, Table S2), to simulate a strong northward shift of the monsoon up to the Mediterranean area (Fig. 3, *2a*). In simulation 3 (Fig. 3, *3a*), the 300 mm were mostly distributed at latitudes lower than 18° N in the JJA months and mostly at latitudes higher than 24° N in the DJF months (November to February) and between 18 and 24° N in both JJA and DJF (transition zone) (Fig. 3, *3a* and *SI Appendix*, Table S2). CARAIB was run with these two 30-y series of climatic data in a 9-ka configuration (263 ppmv of CO_2_ and 9-ka orbital parameters). Air relative humidity and cloudiness fields were calculated using statistical relationships estimated from observations in the region (see *SI Appendix* for details).

## Supplementary Material

Supplementary File

## Data Availability

All fossil data of Tislit have been deposited in Pangaea (https://doi.pangaea.de/10.1594/PANGAEA.925930) (71). All other study data are included in the article and/or supporting information.

## Change History

July 17, 2021: The Acknowledgments have been updated.
