# Supplementary material for: Early Holocene greening of the Sahara requires Mediterranean winter rainfall
Source: Proc Natl Acad Sci U S A. 2021 May 31;118(23):e2024898118. doi: 10.1073/pnas.2024898118 (PMC8201883; doi:10.1073/pnas.2024898118)
Supplement: Supplementary File [file pnas.2024898118.sapp.pdf]

Supplementary information

(8 figures, 2 tables, links to 16 datasets)

Figures

Figure S1. The chronology of the Tislit core is based on eight radiocarbon dates (blue). The ^{14}C calibrated ages with 95% confidence intervals (black) and the age-depth model were obtained with CLAM software version 2.2[1], using the calibration data set IntCal13[2]. The age-depth model provides a basal age of about 18,500 years cal BP. The accumulation is continuous with a rather constant rate in the whole core. ^{14}C datings were performed by ARTEMIS national facility using accelerator mass spectroscopy.

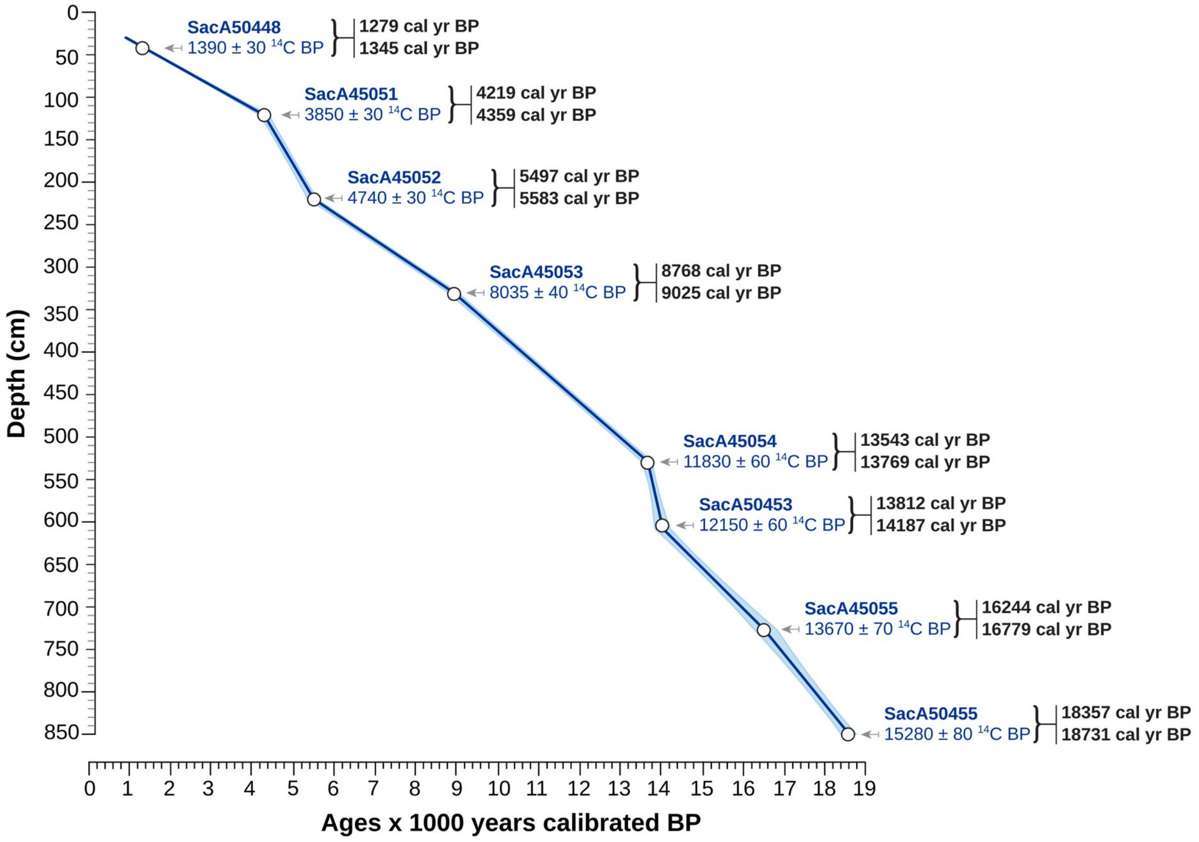

Figure S2: A set of 171 sub-samples, with an average time resolution of about 100 years, were treated chemically to extract their pollen content, using HCl, KOH, $ZnCl_2$ and acetolysis (sulfuric acid and acetic anhydride). Pollen grains were identified and counted using a photonic microscope. We identified 78 pollen taxa, of which 10 were aquatic. The pollen sums and percentages in each sample are based on trees, shrubs and herbs, and exclude aquatic plants and fern spores. The number of pollen grains counted per sample was between 108 and 758 (with a mean of ca. 220). Ten samples were pollen barren, 7 of which were in the uppermost section (0 to 35 cm). Thus, the uppermost pollen sample is dated at ca. 1000 years cal. BP. Pollen abundances of 16 main taxa among the 68 terrestrial taxa identified in the fossil record showing the main dynamics and ecosystem composition over the last 18500 years around the Tislit lake and the clear expansion of the Mediterranean arboreal elements during the African Humid Period (orange shaded).

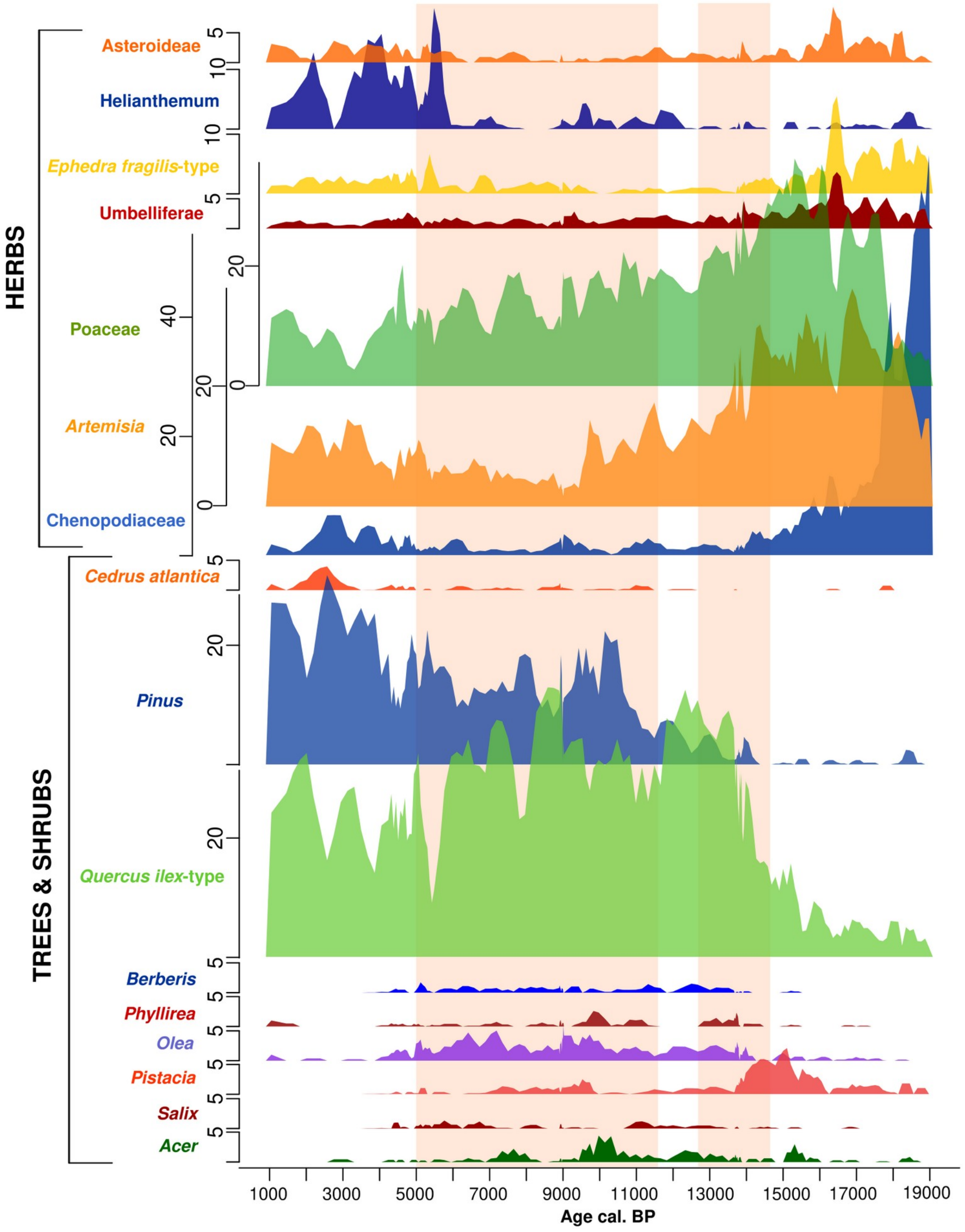

Figure S3: Mean monthly precipitation (mm) of five main Mediterranean plant taxa identified in the Tislit pollen record. The plot shows a marked seasonal distribution of the precipitation throughout the year with clear wet winters and dry summers.

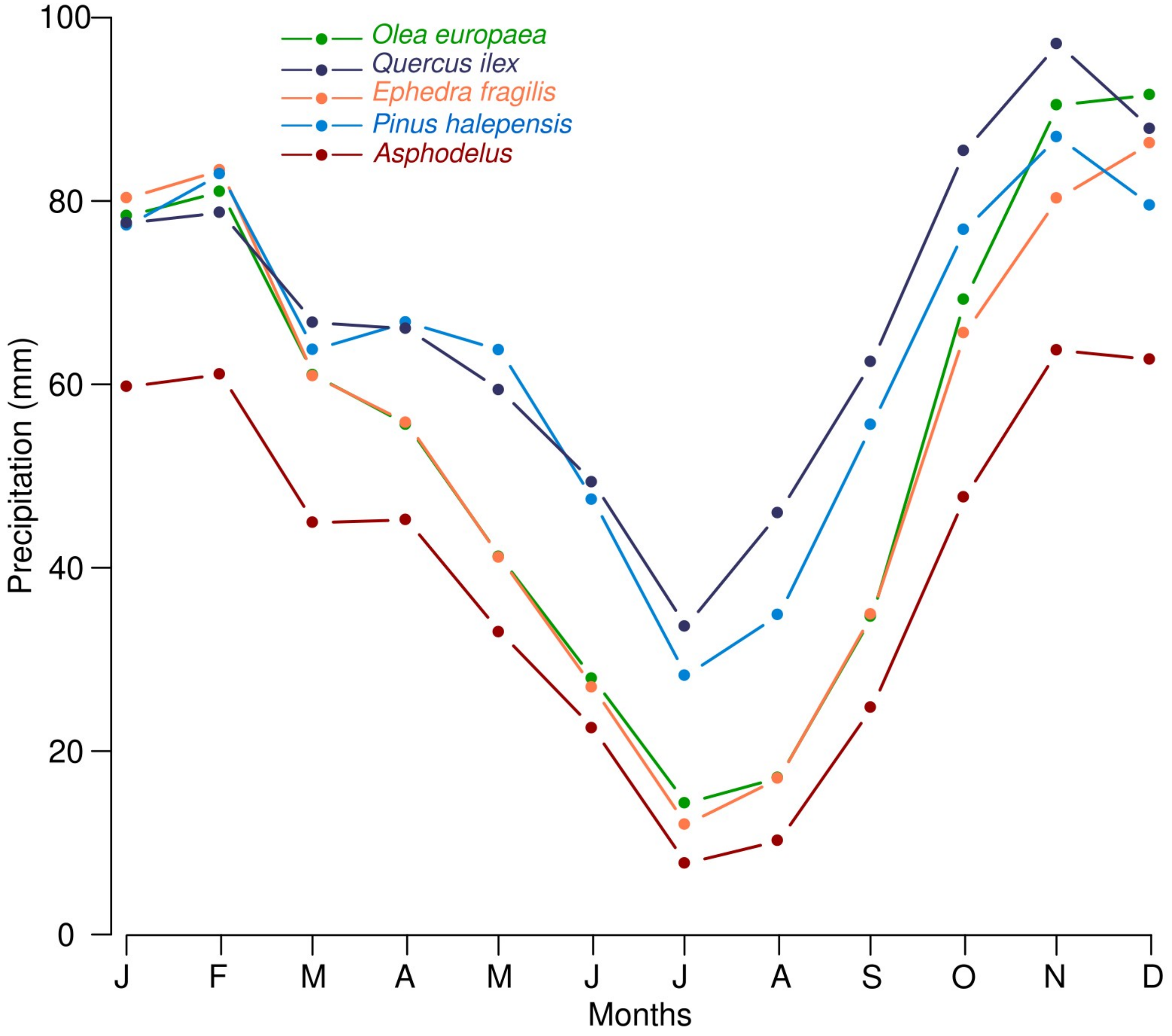

Figure S4: January and July reconstructed temperature changes from the Tislit pollen record. The red marks on the Y axes indicate the modern values in the Imilchil closest weather station. The shaded blue area corresponds to the African Humid period.

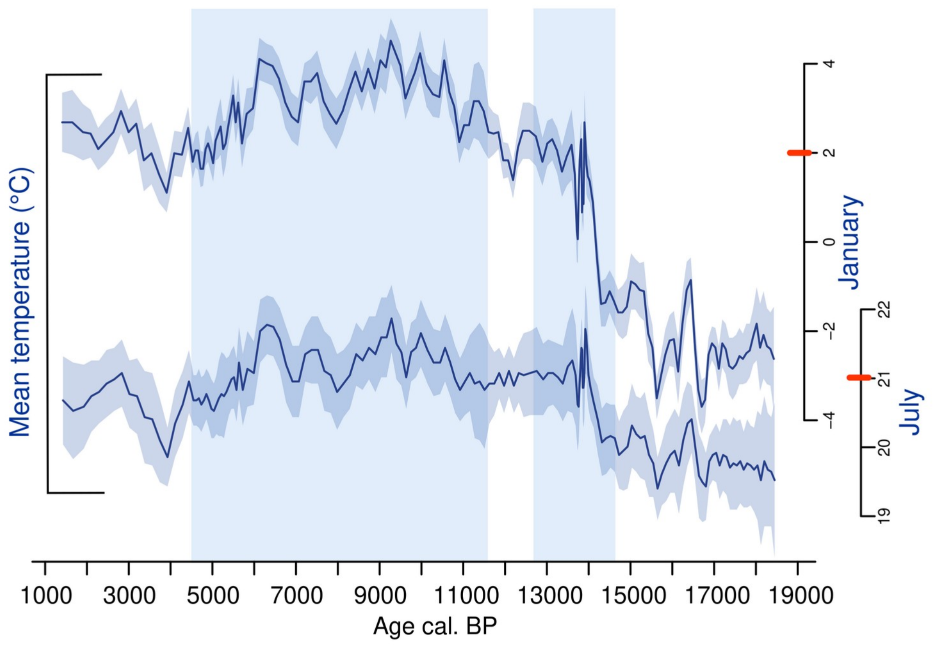

Figure S5: The map shows the location of modern pollen samples[3] (https://github.com/EMPD2/EMPD-data) (green dots) and modern gridded dataset from WORLDCLIM dabatase[4] (red crosses). The red rectangle on the map corresponds to the climatic cell where Lake Tislit is located. Plots show Pearson coefficient correlations (r) between annual and seasonal precipitation (Winter (DJF), Spring (MAM) and Summer (JJA)) reconstructed from the modern pollen dataset and WORLDCLIM gridded precipitation values. All p-values are lower than 1%. The red dots on the regression plots correspond to precipitation values of a WORLDCLIM grid (-5.25, 32.25) and a modern pollen sample (-5.05, 32.17) which is the closest to the location of Lake Tislit (-5.38, 32.11). The standard error estimated from this validation dataset is ±29, ±15, ±8, and ±52 mm/day for winter, spring, summer and annual precipitation, respectively. It should be noted that this dataset overestimates the method uncertainty because it is based on soil samples instead of lake sediment samples. The uncertainty in our method is rather estimated by its sensitivity to the occurrence of individual taxa using a jack-knife approach, explained in the materials and method section. The average uncertainty from the jack-knife approach is ±41, ±22 , ±33 , and ±60 mm/day for annual, spring, summer, and winter precipitation respectively.

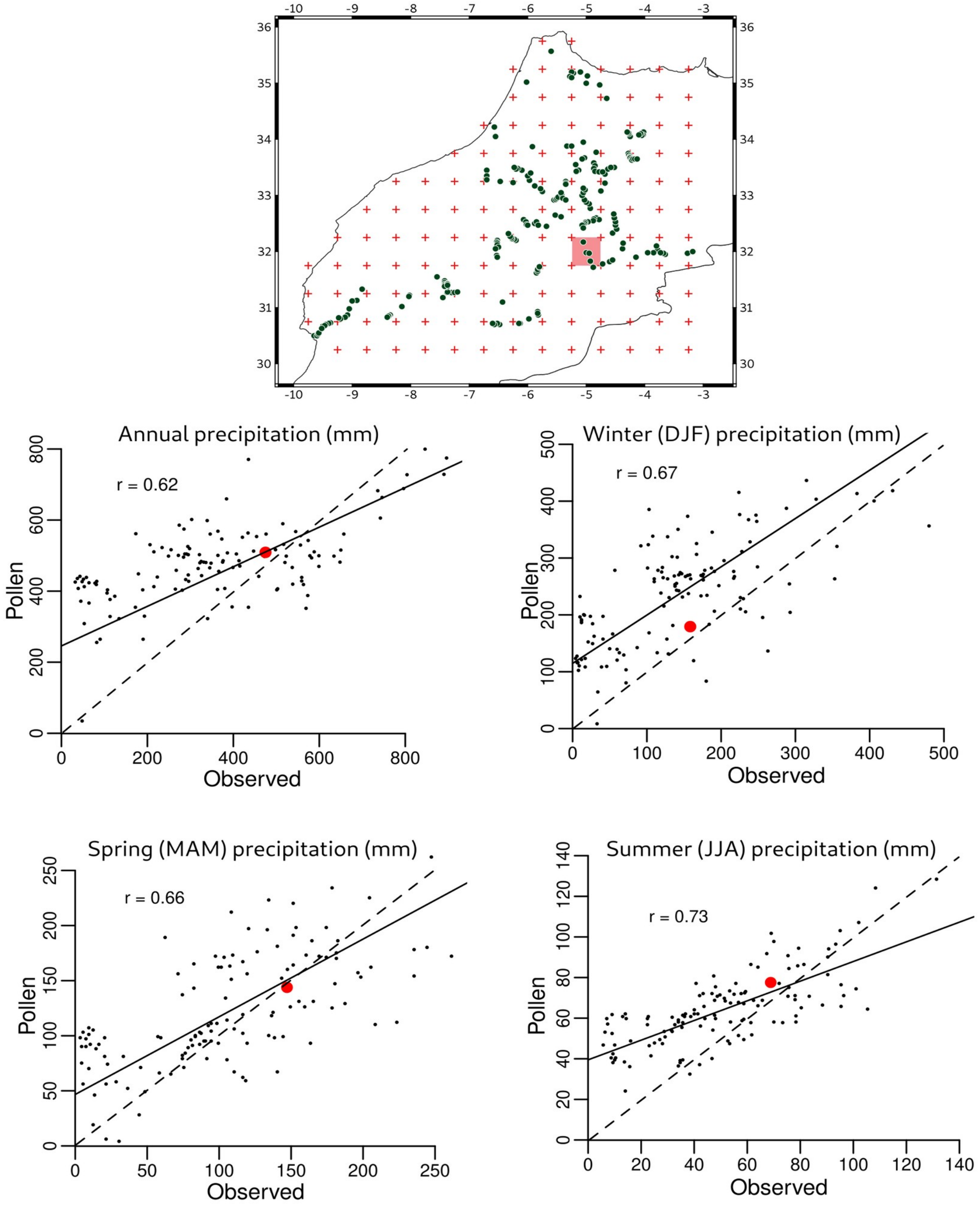

Leaf wax analyses

$\delta^{13}C$ analyses of *n*-alkanes were conducted on a ThermoFisher Scientific MAT 252 isotope ratio mass spectrometer that was coupled, via a GC-C combustion interface with a nickel catalyzer operating at 1000 °C, to a ThermoFisher Scientific Trace GC equipped with a HP-5ms column (30 m, 0.25 mm, 0.25 µm). Each sample was measured in duplicate if sufficient material was available. $\delta^{13}C$ values were calibrated against a CO_2 reference gas of known isotopic composition, and are given in ‰ VPDB. Accuracy and precision were determined by measuring *n*-alkane standards, calibrated against the A4-Mix isotope standard (A. Schimmelmann, University of Indiana) every six measurements. The difference between the long-term means and the measured standard values yielded a 1σ error of <0.3 ‰. The accuracy and precision of the squalane internal standard were both 0.1 ‰. The precision of the replicate analyses of the *n*-C_{29} and *n*-C_{31} alkanes was 0.2 and 0.1 ‰ on average, respectively (<0.1 – 0.7 ‰ for *n*-C_{29} and <0.1 – 0.6 ‰ for *n*-C_{31} alkane). For samples which could only be analyzed once, the long-term precision of the standards (0.3 ‰) was assumed. As $\delta^{13}C$ values of the *n*-C_{29} and *n*-C_{31} alkanes co-vary strongly (r=0.81), we calculated a weighted average $\delta^{13}C$ value for $\delta^{13}C_{wax}$ using their relative abundance. $\delta^{13}C_{wax}$ varies from -33.3 to -24.5 ‰ VPDB, with an average propagated precision of 0.2 ‰ (<0.1 – 0.7). Mostly, $\delta^{13}C_{wax}$ showed depleted ^{13}C values, indicative of C3 plant vegetation, with the exception of the period corresponding to Heinrich event 1 (HE1, 16.1 – 14.9 ka BP), during which there were enriched ^{13}C values (figure S6). This is consistent with the natural vegetation in Morocco, which is entirely of the C3 type in modern conditions. The elevated $\delta^{13}C_{wax}$ values during HE1 coincide with an abundance of Poaceae and could thus indicate an expansion of C4 grasses around Lake Tislit under the extreme climatic conditions of HE1.

δD analyses of *n*-alkanes were conducted on a Thermo Fisher Scientific MAT 253™ Isotope Ratio Mass Spectrometer that was coupled, via a GC IsoLink operating at 1420°C, to a Thermo Fisher Scientific TRACE GC equipped with a HP-5ms column (30 m, 0.25 mm, 1 µm). Each sample was measured in duplicate if sufficient material was available. δD values were calibrated against an H_2 reference gas of known isotopic composition and are given in ‰ VSMOW. Accuracy and precision were controlled by the laboratory's internal *n*-alkane standard, calibrated against the A4-Mix isotope standard every six measurements, and by daily determination of the H_3^+ factor. Measurement precision was determined by calculating the difference between the analyzed values of each standard measurement and the long-term mean of standard measurements, which yielded a 1σ error of <3 ‰. H_3^+ factors varied between 5.0 and 5.2. The accuracy and precision of the squalane internal standard were 4 ‰ and 3 ‰, respectively. The precision of the replicate analyses of the *n*-C_{29} and *n*-C_{31} alkanes was 1 ‰ on average (<1 – 5 ‰). For samples which could only be analyzed once, the long-term precision of the standards (3 ‰) was assumed. As δD values of the *n*-C_{29} and *n*-C_{31} alkanes co-vary (r=0.78), we calculated a weighted average δD value for $\delta D_{wax,}$ using their relative abundance. δD_{wax} varies between -162 to -193 ‰ VSMOW, with an average propagated precision of 2 ‰ (<1 – 5 ‰) (figure S6). As different vegetation types can cause offsets in δD_{wax}, due to different hydrogen isotope fractionation factors, we estimated δD_{precip} using $\delta^{13}C_{wax}$ as an estimate for vegetation type, according to established procedures[5]. For comparability, we used $\delta^{13}C_{wax}$ end-members and fractionation factors reported by Tierney[6] and based on Garcin[7] and Sachse[8]. δD_{precip} estimates range from -54 to -87 ‰ VSMOW (figure 5 in SI), which are consistently more depleted than the δD_{precip} estimates for GC27[6]. This is due to the high altitude setting of Lake Tislit and reflects the input of plant waxes from local sources. Additionally, the δD_{precip} estimates were compensated for global ice volume changes affecting isotopes in the hydrological cycle. For this purpose, Bintanja's marine oxygen isotope compilation[9] was used, with the LGM-present $\delta^{18}O$ range scaled to 1 ‰ VSMOW. This ice-volume correction has an insignificant effect for the Holocene and

leads to slightly depleted δD_{precip} estimates for the period over 10,000 years ago (figure S6). In addition, we compensated the δD_{precip} estimates for temperature changes, as changes in condensation temperature lead to changes in δD_{precip}[76]. In the absence of a specific local temperature lapse rate for Morocco, we used the global average of 5.6 ‰/°C for δD_{precip}[10]. We used the averaged winter temperature (Dec - Feb) from the pollen reconstructions. The essential effect of the temperature compensation is more enriched δD_{precip} estimates when winter temperatures were lower, i.e. prior to the Holocene (figure S6).

Figure S6: Stable isotope compositions of plant wax in Tilsit core. a) $\delta^{13}C$ compositions of the *n*-C_{29} (blue) and *n*-C_{31} alkane (orange) and weighted average $\delta^{13}C_{wax}$ (green), b) δD compositions of the *n*-C_{29} (blue) and *n*-C_{31} alkane (orange) and weighted average δD_{wax} (green), c) δD_{precip} estimates (see main text) and effects of ice volume correction ($\delta D_{precip-ic}$) and temperature correction ($\delta D_{precip-ic-tc}$). Data in c) are shown without error estimates to compare effects of corrections.

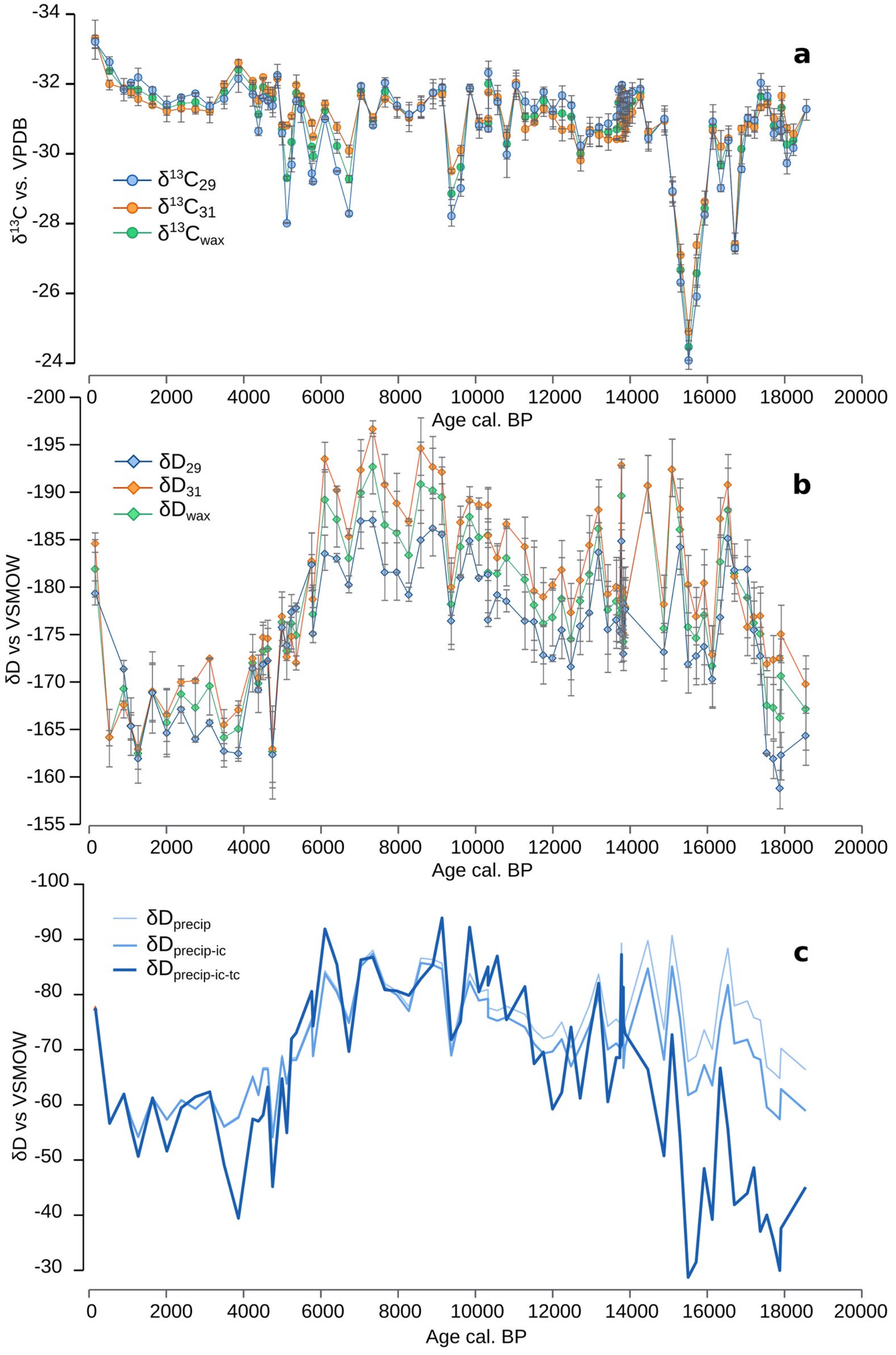

Vegetation model simulations

CARAIB is a mechanistic model that describes stomatal regulation and photosynthesis, growth and respiration, competition for resources, and mortality of a set of plant objects, which can be plant functional types (PFT), bioclimatic affinity groups or plant species. The daily model inputs are mean air temperature T, diurnal thermal amplitude Tmax - Tmin, precipitation P, percentage of sunshine hours SH, air relative humidity RH, and wind speed W. The main model outputs are soil water amount and water fluxes, as well as the abundance, gross and net primary productivity (GPP and NPP), biomass and leaf area index of all PFTs. From these outputs, a biome map can be constructed in a post-processing subroutine[11-13].

PFTs have been used in accordance with Henrot's classification[14], which includes 26 PFTs designed for large-scale or global applications. These PFTs are distributed between two vegetation storeys: grasses and shrubs (PFTs 1-11) in the understorey, and trees (PFTs 12-26) in the overstorey. The model also calculates a budget of water fluxes to evaluate the amount of soil water in the root zone. Soil water deficit can induce: (1) stomatal closure and reduction of photosynthetic assimilation; (2) reduction of the leaf area index (leaf desiccation); and, under most severe water stress, (3) mortality of the PFTs. Natural fires can also induce mortality during dry periods. The model time step is 1 day for updating soil water or carbon reservoirs, and 2 hours for the calculation of photosynthesis and respiration fluxes.

In simulation 1 (figure 3.1), we selected the HadCM3 general circulation model[15], as it performed an almost transient simulation over the last 21,000 years (with 1,000-year snapshots), with atmosphere and ocean grids of 3.75° x 2.5° and 1.25° x 1.25°, respectively, over 20 levels each. For the 9 ka experiments of HadCM3, only monthly mean data were available. The monthly anomalies were then calculated as the absolute anomaly (i.e., the difference between the 9 ka simulation and the pre-industrial control simulation) for temperature, and as a weighted mean of the absolute and relative (i.e., the same difference between the 9 ka simulation and the control, multiplied by the ratio between the observed and the control simulation values) anomaly for precipitation. The weights were chosen in such a way that they favored the absolute anomaly whenever possible, except when negative precipitation tended to be produced for 9 ka. In the latter situation, the weight of the relative anomaly was progressively increased[16]. The monthly anomalies were then simply added to the 1901-1930 monthly mean values, yielding a 30-year sequence of monthly temperature and precipitation for 9 ka. CARAIB's weather generator was then used to produce the daily values necessary to force the simulations for each year, drawing on the monthly sequence. This weather generator is based on the currently observed day-to-day variability of temperature (minimum and maximum) and precipitation per (geo-)climatic zone. A renormalization process is performed to ensure that the monthly values are not altered[17]. Note that the day-to-day variability reconstructed by the generator differs from that of the GSWP3 1901-1930 signal. However, this should not significantly impact the results, since both variabilities are based on meteorological data collected in a similar climatic zone (hence with similar distribution functions), and a relatively long record (30 years) is generated.

For consistency, in simulations 2 and 3 (figure 3.2 and 3.3), the air relative humidity r was increased and the percentage of sunshine hours s was decreased at the same time as the precipitation was increased. These changes in r and s are critical for maintaining a Green Sahara, since they significantly reduce evapotranspiration and thus lead to wetter soils. They were estimated on the basis of statistical relationships established in the simulated areas for the 1901-1930 period in our climate dataset.

Using the monthly climatological means for 1901-1930, a significant linear correlation was indeed obtained in a log-log plot of relative humidity r (or sunshine hours s) versus precipitation that was increased by 1 mm/month (P+1). The Pearson correlation coefficient was 0.772 for r and -0.759 for s, when all months and all pixels of the simulated domain were included. This yielded the following relationships, allowing us to calculate r and s as a function of precipitation P:

$$r(P)=\alpha_r \exp[\beta_r \ln(P+1)] \qquad (5 < r < 100)$$

$$s(P)=\alpha_s \exp[\beta_s \ln(P+1)] \qquad (0 < s < 100)$$

where r and s are expressed in % and limited to the indicated ranges, precipitation P is in mm/month, and α_r = 23,0233, β_r = 0,2153, α_s = 88,8467, β_s = - 0,0936193. These relationships allowed us to calculate the anomalies:

$$\Delta r=r(P_{9ka})-r(P_{0ka})$$

$$\Delta s=s(P_{9ka})-s(P_{0ka})$$

to be added to each value of r and s in the 1901-1930 time series, to obtain a 30-year time series of r and s for simulations 2 and 3.

Figure S7: Control simulation of the model CARAIB run with the pre-industrial atmospheric CO_2 (280 ppmv). After a spin-up phase, the model was run with the meteorological dataset that covers years 1901-1930 (see materials and methods section). The plotted biomes correspond to an average of the simulations over 30 years. The simulated biome distribution is quite consistent with observed modern vegetation in northern Africa (compare with, e.g., Sayre et al., 2013. A New Map of Standardized Terrestrial Ecosystems of Africa. Washington, DC: Association of American Geographers. 24 pages).

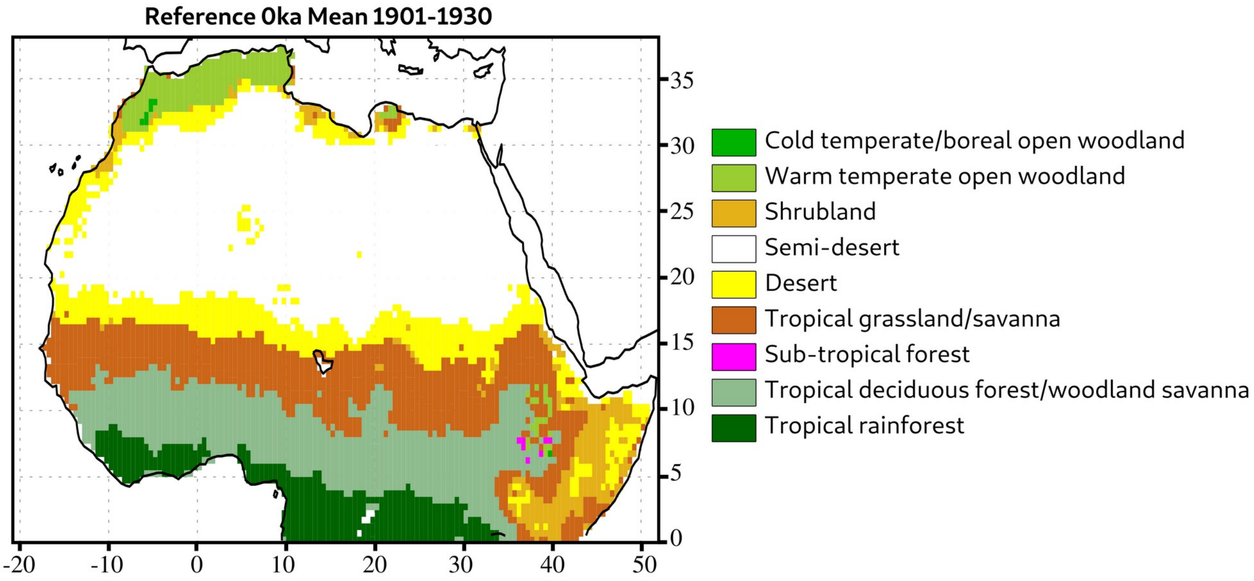

Figure S8: Precipitation amplitude (median, first and fourth quartiles) of 45 taxa used in the pollen-based reconstruction from the Tislit record. The 45 pollen taxa are plotted in an increasing order of precipitation values.

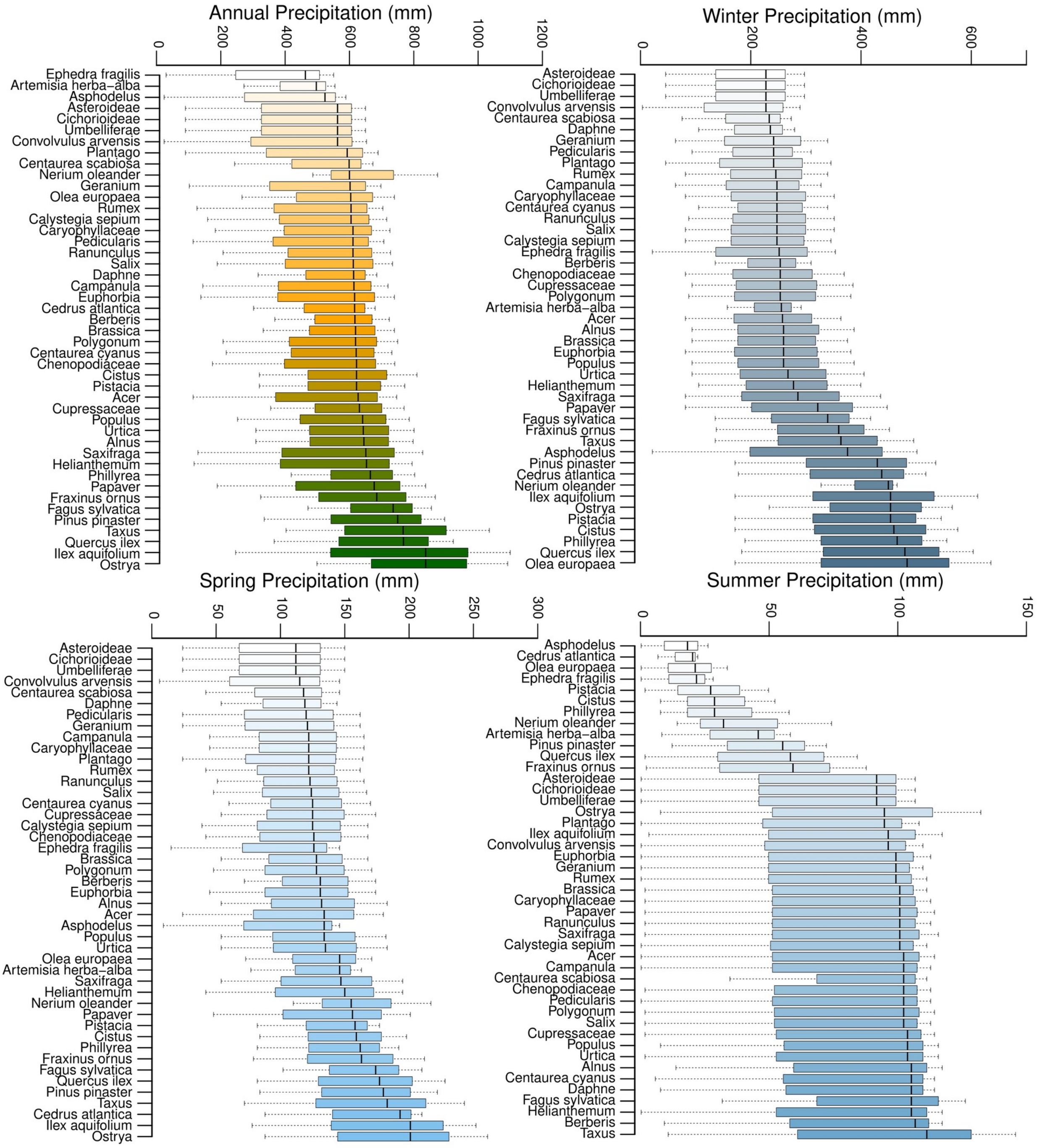

Tables

Table S1. Identified pollen taxa of trees, shrubs and herbs in the Tislit record (68 taxa) with those used for the pollen-based climate reconstruction (45 in bold character).

Acer	**Chenopodiaceae**	*Illecebrum*	***Populus***
Alnus	**Cichorioideae**	***Ilex aquifolium***	Primulaceae
Macrochloa tenacissima	***Cistus***	Lamiaceae	***Quercus ilex***
**Umbelliferae**	***Convolvulus arvensis***	***Linum***	*Rhus*
Artemisia herba-alba	***Ostrya***	Liliaceae	Rosaceae
Asphodelus	**Cupressaceae**	*Malva sylvestris*	Rubiaceae
Asteroideae	***Daphne***	***Nerium oleander***	***Rumex***
Berberis	*Digitalis*	***Olea europaea***	***Ranunculus***
Boraginaceae	***Ephedra fragilis***	***Papaver***	***Salix***
Brassica	*Eucalyptus*	***Pedicularis***	*Salvadora*
Calystegia sepium	***Euphorbia***	*Scrophularia*	***Saxifraga***
Campanula	*Fabaceae*	***Phillyrea***	***Centaurea scabiosa***
**Caryophyllaceae**	***Fagus sylvatica***	***Pinus pinaster***	***Taxus***
Castilleja fissiflolia	***Fraxinus ornus***	***Pistacia***	Ulmaceae
Cedrus atlantica	***Geranium***	***Plantago***	***Urtica***
Centaurea cyanus	***Helianthemum***	Poaceae	*Veronica*
Cereals	*Hibiscus*	***Polygonum***	*Vitis*

Table S2: Percentages of the progressive additional 300mm precipitation to the modern monthly values in simulation 3, over five latitudinal belts between 0°N and 36°N.

**Latitude**	**Jan**	**Feb**	**Mar**	**Apr**	**Mai**	**Jun**	**Jul**	**Aug**	**Sep**	**Oct**	**Nov**	**Dec**
**>0°N, <18°N**	0.05	0.05	0.05	0.05	0.05	0.1	0.2	0.2	0.1	0.05	0.05	0.05
**>18°N, <=20°N**	0.1	0.05	0.05	0.05	0.05	0.1	0.15	0.15	0.1	0.05	0.05	0.1
**>20°N, <=22°N**	0.125	0.1	0.05	0.05	0.05	0.05	0.1	0.15	0.05	0.05	0.1	0.125
**>22°N, <=24°N**	0.175	0.1	0.05	0.05	0.05	0.05	0.05	0.1	0.05	0.05	0.1	0.175
**>24°N, <36°N**	0.2	0.1	0.05	0.05	0.05	0.05	0.05	0.05	0.05	0.05	0.1	0.2

Data availability

All datasets may be downloaded from the database PANGAEA at https://doi.pangaea.de/10.1594/PANGAEA.925930

D1 - Modern monthly precipitation with additional 300mm all throughout Northern Africa, distributed over the summer months, used for the vegetation model simulation in figure 2a. (prcMEAN1901_1930.dat)
Authors: Louis François and Rachid Cheddadi
Deposited: May 1st, 2021

D2 - Modern monthly precipitation with additional 300mm for the vegetation model simulation in figure 3a. This dataset includes additional 300mm precipitation below 18°N during the summer season, above 24°N during the winter season, and a progressive transition of the additional 300mm between 18°N and 24°N (see table S2). (prcMEAN1901_1930.dat)
Authors : Louis François and Rachid Cheddadi
Deposited: May 1st, 2021

D 3 - Simulated biomes values using HADCM3 climatology (figure 1b) (biomeMEAN1901_1930.res)
Author : Louis François
Deposited: May 1st, 2021

D4 - Simulated NPP values using HADCM3 climatology (figure 1c). (nppmthMEAN1901_1930.res)
Author : Louis François
Deposited: May 1st, 2021

D5 – Simulated biomes values using precipitation scenario for the second CARAIB simulation (figure 2b). (biomeMEAN1901_1930.res)
Author : Louis François
Deposited: May 1st, 2021

D6 – Simulated NPP values using precipitation scenario for the second CARAIB simulation (figure 2c). (nppmthMEAN1901_1930.res)
Author : Louis François
Deposited: May 1st, 2021

D7 - Simulated biomes and NPP values using precipitation scenario for the third CARAIB simulation (figure 3b). (biomeMEAN1901_1930.res)
Author : Louis François
Deposited: May 1st, 2021

D8 - Simulated NPP values using precipitation scenario for the third CARAIB simulation (figure 3c). (nppmthMEAN1901_1930.res)
Author : Louis François
Deposited: May 1st, 2021

D9 - Modern pollen data set used for reconstructing the modern precipitation in Morocco (figure S5). (surf_data.csv)
Author : Rachid Cheddadi
Deposited: May 1st, 2021

D10 - Modern pollen-based reconstruction of precipitation (figure S5). (surf_prc.csv)
Author : Rachid Cheddadi
Deposited: May 1st, 2021

D11 - Lipids and stable isotopes
Authors: Enno Schefuß and Majda Nourelbait
Deposited: November 1st, 2021

D12 - Oxygen isotopes from ostracod shells
Author : Matthieu Carré
Deposited: November 1st, 2021

D13 - Pollen analyses
Author : Majda Nourelbait
Deposited: November 1st, 2021

D14- Precipitation pollen-based reconstruction
Author : Rachid Cheddadi
Deposited: November 1st, 2021

D15- Temperature pollen-based reconstruction
Author : Rachid Cheddadi
Deposited: November 1st, 2021

D16- Radiocarbon dates
Author: LMC14, Laboratoire de Mesure du Carbone-14, ARTEMIS national facility. Contributed by Rachid Cheddadi
Deposited: November 1st, 2021

References

1. M. Blaauw, Methods and code for 'classical'age-modelling of radiocarbon sequences. *Quaternary geochronology* 5, 512-518 (2010).
2. P. J. Reimer et al., IntCal13 and Marine13 radiocarbon age calibration curves 0–50,000 years cal BP. *Radiocarbon* 55, 1869-1887 (2013).
3. R. Cheddadi, H. Lamb, J. Guiot, S. van der Kaars, Holocene climatic change in Morocco: a quantitative reconstruction from pollen data. *Climate dynamics* 14, 883-890 (1998).
4. R. J. Hijmans et al., Very high resolution interpolated climate surfaces for global land areas. *International Journal of Climatology: A Journal of the Royal Meteorological Society* 25, 1965-1978 (2005).
5. A. Collins *et al.*, Estimating the hydrogen isotopic composition of past precipitation using leaf-waxes from western Africa. *Quaternary Science Reviews* 65, 88-101 (2013).
6. J. E. Tierney, F. S. Pausata, P. B. deMenocal, Rainfall regimes of the Green Sahara. *Science advances* 3, 1-10 (2017).
7. Y. Garcin *et al.*, Reconstructing C3 and C4 vegetation cover using n-alkane carbon isotope ratios in recent lake sediments from Cameroon, Western Central Africa. *Geochimica et Cosmochimica Acta* 142, 482-500 (2014).
8. D. Sachse et al., Molecular paleohydrology: interpreting the hydrogen-isotopic composition of lipid biomarkers from photosynthesizing organisms. *Annual Review of Earth and Planetary Sciences* 40, 221-249 (2012).
9. E. Bray, E. Evans, Distribution of n-paraffins as a clue to recognition of source beds. *Geochimica et Cosmochimica Acta* 22, 2-15 (1961).
10. W. Dansgaard, Stable isotopes in precipitation. *Tellus* 16, 436-468 (1964).
11. P. Warnant, L. François, D. Strivay, J. C. Gérard, CARAIB: a global model of terrestrial biological productivity. *Global biogeochemical cycles* 8, 255-270 (1994).
12. J.-C. Gérard, B. Nemry, L. Francois, P. Warnant, The interannual change of atmospheric CO2: Contribution of subtropical ecosystems? *Geophysical Research Letters* 26, 243-246 (1999).
13. M. Dury *et al.*, Responses of European forest ecosystems to 21 (st) century climate: assessing changes in interannual variability and fire intensity. *iForest: Biogeosciences and Forestry* 4, 82-99 (2011).
14. A.-J. Henrot *et al.*, Middle Miocene climate and vegetation models and their validation with proxy data. *Palaeogeography, Palaeoclimatology, Palaeoecology* 467, 95-119 (2017).
15. J. S. Singarayer, P. J. Valdes, High-latitude climate sensitivity to ice-sheet forcing over the last 120 kyr. *Quaternary Science Reviews* 29, 43-55 (2010).
16. L. François, M. Ghislain, D. Otto, A. Micheels, Late Miocene vegetation reconstruction with the CARAIB model. *Palaeogeography, Palaeoclimatology, Palaeoecology* 238, 302-320 (2006).
17. B. Hubert, L. François, P. Warnant, D. Strivay, Stochastic generation of meteorological variables and effects on global models of water and carbon cycles in vegetation and soils. *Journal of hydrology* 212-213, 318-334 (1998).
